# Motor Ingredients Derived from a Wearable Sensor-Based Virtual Reality System for Frozen Shoulder Rehabilitation

**DOI:** 10.1155/2016/7075464

**Published:** 2016-08-23

**Authors:** Si-Huei Lee, Shih-Ching Yeh, Rai-Chi Chan, Shuya Chen, Geng Yang, Li-Rong Zheng

**Affiliations:** ^1^Department of Physical Medicine and Rehabilitation, Taipei Veterans General Hospital and National Yang-Ming University, Taipei, Taiwan; ^2^School of Information Science and Technology, Fudan University, Shanghai, China; ^3^Department of Physical Therapy, China Medical University, Taichung, Taiwan

## Abstract

*Objective*. This study aims to extract motor ingredients through data mining from wearable sensors in a virtual reality goal-directed shoulder rehabilitation (GDSR) system and to examine their effects toward clinical assessment.* Design*. A single-group before/after comparison.* Setting*. Outpatient research hospital.* Subjects*. 16 patients with frozen shoulder.* Interventions*. The rehabilitation treatment involved GDSR exercises, hot pack, and interferential therapy. All patients first received hot pack and interferential therapy on the shoulder joints before engaging in the exercises. The GDSR exercise sessions were 40 minutes twice a week for 4 weeks.* Main Measures*. Clinical assessments included Constant and Murley score, range of motion of the shoulder, and muscle strength of upper arm as main measures. Motor indices from sensor data and task performance were measured as secondary measures.* Results*. The pre- and posttest results for task performance, motor indices, and the clinical assessments indicated significant improvement for the majority of the assessed items. Correlation analysis between the task performance and clinical assessments revealed significant correlations among a number of items. Stepwise regression analysis showed that task performance effectively predicted the results of several clinical assessment items.* Conclusions*. The motor ingredients derived from the wearable sensor and task performance are applicable and adequate to examine and predict clinical improvement after GDSR training.

## 1. Introduction

Frozen shoulder is considered a common intrinsic shoulder dysfunction that affects patients' daily living activities (ADL). Aside from medication, physical therapy is also needed to effectively restore shoulder joint functionality [1]. A crucial criterion for successful physical therapy involves the appropriate use of exercise, such as range of motion (ROM) exercises, stretching [2], pulley therapy, wall-climbing exercises involving the frontal and sagittal movement planes, exercises using towels, and joint mobilization, to sever capsule adhesions and improve joint mobility and performance. In addition, sufficient amount of muscle strengthening exercises must be provided to prevent muscle atrophy that results from a lack of exercise over an extended period.

With advancements in technology, researchers have begun to adopt virtual reality (VR) techniques in rehabilitation [3–7]. In addition, wearable sensors, based on inertial measurement unit (IMU), can be used to measure the movement and provide immediate feedback to alert patients about the movement errors [8–12]. Previous studies aforementioned mostly employed VR techniques or wearable sensor to provide systems for motor training. However, motor ingredients using data mining technique from the sensor or the task performance during training were not considered as a way to evaluate the therapeutic effect; instead, clinical assessment tools (CATs) were adopted [13, 14].

In this study, we presented an innovative goal-directed shoulder rehabilitation (GDSR) system by combining VR techniques with wearable IMU (WIMU) to design rehabilitation exercises (specifically, shoulder ROM exercises, shoulder muscle strengthening, and core muscle strengthening) for patients with frozen shoulder. Based on the motor training by GDSR, we further proposed a variety of motor ingredients, including motor indices and task performance, extracted from the raw data of WIMU. The motor ingredients might provide clues revealing motor functions. The objective of this study was to investigate the effects of proposed motor indices and task performance on motor functions of shoulder. Therefore, the proposed motor indices and task performance were examined in three ways. (1) Were they significantly improved from pretest to posttest? (2) Were they significantly correlated with clinical assessment items? (3) Which one of them was more influential to the clinical assessment items?

Along with the growth of techniques of internet, cloud-computing, and big-data processing, Internet of Things (IoT) based telerehabilitation was proposed in which VR system was able to be an end-system serving home-based motor training. Beyond that, the data collected from end-system (e.g., WIMU) was further proposed to undergo big-data processing on cloud system in order to provide home-based assessment equivalent to existing clinical assessment tool (e.g., CATs), while clinical assessment was only available in hospitals instead of at home. More importantly, the assessment results might be a reference for the plan of daily training program. As a result, this was a pilot study to investigate the relevance between the data collected from WIMU and the clinical assessment items. The results of this study might contribute to understanding the feasibility of WIMU data intended to apply to home-based motor assessment.

## 2. Methods and Materials

### 2.1. Participants

In this experiment, we recruited 16 patients with frozen shoulders who met the inclusion criteria. The inclusion criteria were as follows: patients who (1) were over 20 years of age; (2) never received physical therapy; (3) exhibited normal cognition and could follow instructions when using the system; (4) were clinically confirmed to have frozen shoulders; and (5) had signed a consent form. The exclusion criteria were as follows: patients who (1) had history of a proximal humeral fracture or dislocation of glenohumeral joint; (2) received hyaluronic acid injection in the shoulder; (3) experienced cervical radiculopathy or had been diagnosed with shoulder degenerative joint diseases; (4) were at the final stage of a malignant disease; or (5) were pregnant. Overall, 6 males and 10 women satisfied the required conditions. The mean age was 58.6 years, and the average duration of injury was 22.7 months.

### 2.2. Interactive VR System

**Figure 1:**
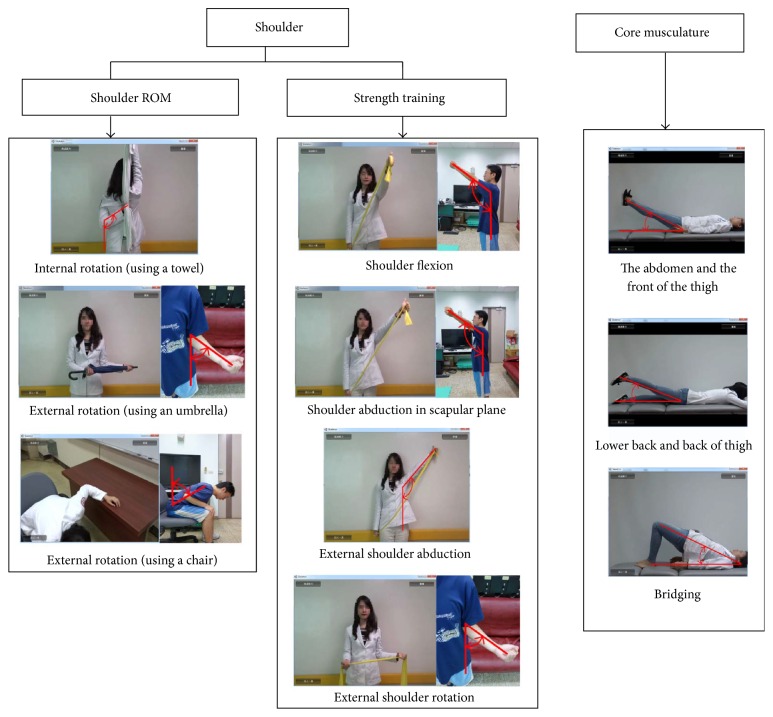
The rehabilitation tasks.

**Figure 2:**
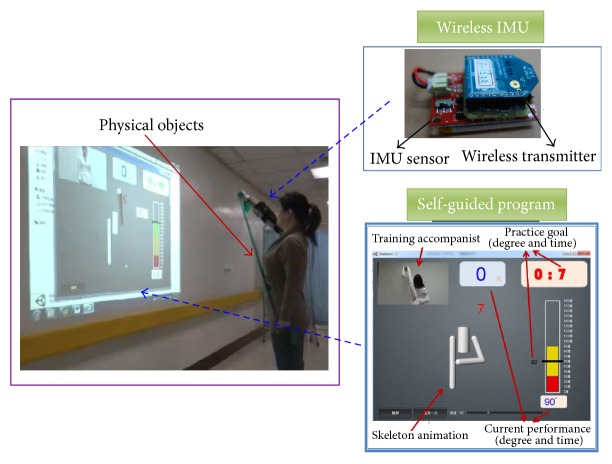
The system architecture.

We designed an interactive motor training system involving shoulder joint stretching, shoulder muscle strengthening, and core muscle strengthening. The unity 3D game-engine software was adopted to formulate a goal-directed shoulder rehabilitation (GDSR), which assists patients with motor rehabilitation. Concurrently, the WIMU sensors were secured to the shoulder joints to measure and record shoulder ROM while patients were undergoing various exercises (see Figure 1). The overall system architecture is presented in Figure 2. In each rehabilitation task, patients were instructed to meet or exceed the target angle predetermined by clinicians and maintain that posture for at least 10 seconds to be considered as successfully completing the task. The GDSR featured a hierarchy of challenges, in which the difficulty level was determined by the target angles set for the rehabilitation tasks and by the number of sets required for each task.

### 2.3. Experimental Procedures

**Figure 3:**
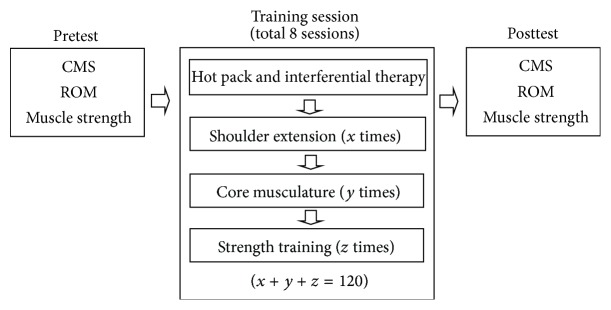
Experimental procedures.

The shoulder rehabilitation involved GDSR exercises, hot pack, and interferential therapy. All patients first received hot pack and interferential therapy on the shoulder joints before engaging in each exercise session. The exercise sessions were 40 minutes twice a week for 4 weeks. Patients were required to employ the GDSR system and complete a series of exercises using various physical objects. The exercise protocol practiced by the patients was as follows: shoulder ROM exercises, shoulder muscle strengthening, and core muscle strengthening. The practice time for each exercise was based on patients' progress and was determined by the therapist. However, the total number of practice for three types of exercise was around 120 times. In addition, before and after the entire rehabilitation course, an independent therapist performed clinical assessments including the Constant-Murley score (CMS), ROM of active and passive shoulder movements, and upper arm muscle strength. Based on patients' severity of injury and their rehabilitation progress, the therapists adjusted the rehabilitation goal in the GDSR system (i.e., the target angles and the total number of exercise repetitions). The experimental procedures are shown in Figure 3.

### 2.4. Outcome Measures

In this study, we used CATs, task performances, and motor indices to examine the effect of shoulder VR rehabilitation system.

#### 2.4.1. CATs

**Figure 4:**
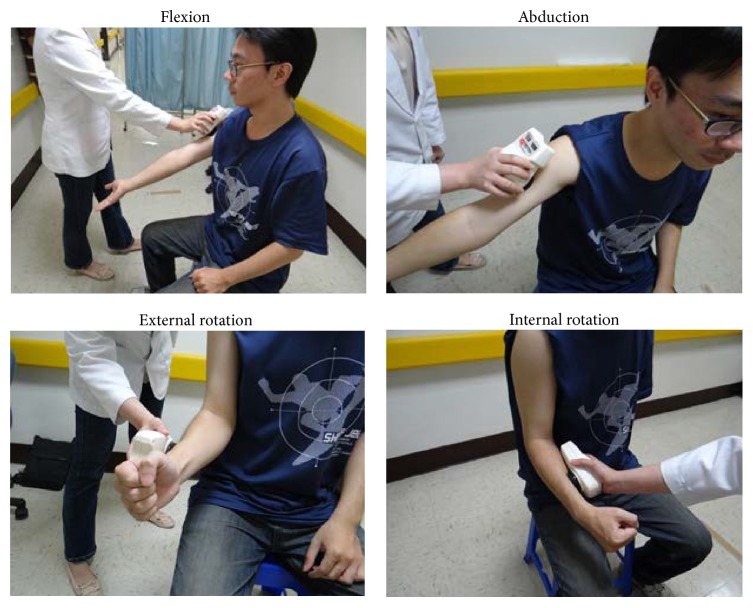
Measurement of upper arm muscle strength.

The CATs were the CMS assessment [15], active and passive shoulder ROM (flexion, abduction, internal rotation, and external rotation) measurement, and upper arm muscle strength test. The CMS assessment was conducted by a therapist not involved in the patient training. Active and passive shoulder ROM were measured using the goniometer in the unit of degree. Arm muscle strength was measured by muscle testing device (microFET3*™*) in the unit of kg (see Figure 4).

#### 2.4.2. Task Performance

Task performance was defined as the patients' abilities to complete the VR tasks in the GDSR system. Task performance was determined based on ROM goal achievable by patients and the ability to maintain postures for 10 seconds and complete a set number of repetitions.

#### 2.4.3. Motor Indices

Based on data extracted from the IMU sensors, the following motor indices were used to assess the exercise performance.

*Angular Velocity*. The angular velocity was calculated via the angular trajectory time history. An increased angular velocity suggested an improvement of joint movement efficiency. The calculation formula was expressed as follows:

(1) $$\begin{array}{c} \omega =\frac{\Delta \theta }{\Delta t}=\frac{\theta_{i}-\theta_{i-1}}{t_{i}-t_{i-1}}, \end{array}$$

where *ω* is angular velocity, *θ* is angle, and *t* is time.

*Number of Interruptions during Joint Rotation*. When performing the exercises, patients were asked to continuously rotate their joints to meet the target angles and complete the tasks. However, during this process, movement might be interrupted because of limited shoulder joint mobility or endurance. The number of interruptions during joint rotation was calculated via the angular trajectory time history, and it could be used as an indicator for assessing joint mobility or endurance. A decreased number of interruptions during joint rotation signified an improved joint mobility or endurance.

*Varying Rate of Muscle Strength*. Based on the elastic coefficient and the length change of the elastic band stretched by the patients, clinicians first calculated the shoulder muscle strength at each time-point and subsequently determined the force time history of the shoulder muscle strength. By using the force time history, the clinicians calculated the strength change per unit time and used this as an indicator to show the rate varying in muscle strength in order to determine whether the muscle strength was improved. The varying rate in muscle strength equation is expressed as follows:

(2) $$\begin{array}{c} \dot{F}=\frac{\Delta F}{\Delta t}=\frac{F_{i}-F_{i-1}}{t_{i}-t_{i-1}}, \end{array}$$

where *F* is force and *t* is time.

### 2.5. Analysis Methods

Statistical analysis was performed in three ways to investigate whether the task performances and the motor indices were applicable to evaluate the therapeutic effect of rehabilitation as clinical assessment items. First, we used Wilcoxon rank sum test to examine if the difference between pretest and posttest was significant in task performances, motor indices, and clinical assessment items. Second, we performed correlation analysis to determine whether correlations of task performance and motor indices with various clinical assessment items did exist. Third, we utilized stepwise regression analysis to examine the predictability of task performance for the clinical assessment results and to examine which variable dominated the prediction.

## 3. Results

### 3.1. CATs

The three CATs (CMS assessment, active and passive shoulder ROM measurement, and upper arm muscle strength measurement) before and after the GDSR treatment are presented in Table 1. The results all indicated significant improvements (*P* < 0.01).

### 3.2. Task Performance

Task performance included the maximum achievable angle of shoulder joint and the number of exercises completed. Given the small sample size, we used the Wilcoxon signed ranks test to analyze task performance between the first and eighth (last session) rehabilitation sessions. The results in Table 2 indicated that the patients' shoulder ROM and numbers of completed exercise improved significantly after eight rehabilitation sessions over 4 weeks (*P* < 0.05). Thus, the GDSR rehabilitation significantly improved shoulder joint mobility and endurance.

### 3.3. Motor Indices

The motor indices included the angular velocity, the number of interruptions during joint rotation, and the varying rate of muscle strength. We used the Wilcoxon signed ranks test to analyze the motor index between the first and eighth rehabilitation sessions. The results show that, after completing the GDSR treatment, the patients' angular velocity for all exercises increased, the majority of which increased significantly (*P* < 0.05) (Table 3). This finding suggested a significant improvement in joint mobility. The difference in the number of interruptions during joint rotation pre- and posttests was not significant; however, it indicated a downward trend. The varying rate of muscle strength was significantly increased after completing the GDSR treatment (*P* < 0.05). This finding indicated that rehabilitation significantly increased muscle strength.

### 3.4. Correlation Analysis

We investigated the correlation between the clinical assessments and task performances using the aforementioned results. The correlations between the clinical assessments and task performances were examined by the Pearson product-moment correlation coefficients for the differences between the pre- and posttest results.  Table 4 shows the correlation coefficients between the maximum achievable angle of the task performances and the clinical assessments. Significant correlations were observed (1) between the task performance of shoulder flexion and the clinical assessment of external rotation (ROM) and (2) between the task performance of shoulder external rotation (crutch) and the clinical assessment of external rotation (muscle strength) (*P* < 0.05).  Table 5 presents the correlation between the number of exercise repetitions and the clinical assessments. Shoulder flexion, shoulder abduction in scapular plane, and shoulder abduction were all significantly correlated with the clinical assessment of shoulder flexion (ROM) and shoulder external rotation (ROM) (*P* < 0.05). Shoulder internal rotation (towel) was significantly correlated with the clinical assessment of shoulder abduction (muscle strength).

### 3.5. The Predictability of Task Performances for Clinical Assessment Results

We used stepwise regression analysis to further investigate the predictability of task performances for the clinical assessment results. Clinical assessments involved three testing items: CMS, ROM, and muscle strength. ROM and muscle strength were further divided into shoulder flexion, shoulder abduction, and internal and external shoulder rotation. Task performance involved maximum angles for various exercises and the number of exercises completed. The results for the stepwise regression analysis are presented in Tables 6 and 7.

Table 6 reveals that the total number of completed shoulder abduction in the scapular plane had an explained variance of 0.289 in the prediction model of the shoulder flexion ROM. This result showed a statistically significant explanatory power of 28.9% (*F* = 7.107, *P* < 0.05) for the shoulder flexion ROM.

Table 7 shows that, among the various variables for predicting the effectiveness of shoulder external rotation (muscle strength), the maximum achievable angle of shoulder external rotation (crutch) had an explained variance of 0.194. This result indicated a statistically significant explanatory power of 19.4% (*F* = 4.604, *P* = 0.050) for the shoulder external rotation (muscle strength). The estimated results confirmed that the maximum achievable angle could effectively predict the results of shoulder external rotation (muscle strength).

## 4. Discussion

We used the pre- and posttest values obtained from the CATs, task performances, and motor indices to evaluate the therapeutic effects of the GDSR exercises. Most testing items in these three outcome measurements showed significant improvements, as shown in Tables 1 [Table tab2]–3. Therefore, the motor ingredients directly derived from the GDSR system including task performance and motor indices possessed similar ability of examining the intervention effects as the CATs.

Each shoulder task performance (including the number of exercises and the maximum achievable angles) showed significant improvement, as presented in Table 2. This finding indicated that shoulder joint mobility improved significantly, which also signified an increased ability to stretch the resistance bands when performing shoulder strengthening exercises. Therefore, there is a potential that the patients' shoulder muscle strength gradually increased with the progress of rehabilitation. Significant improvements were observed in the core muscle task performance (i.e., the number of exercises and the maximum achievable angles). Moreover, the overall muscle strength training intensified as the number of core muscle exercises increased, which indirectly enhanced the therapeutic effect on shoulder muscle strength.

Table 3 shows that, for the external rotation (chair) and core muscle (bridge) exercises, both the angular velocities and the number of interruptions during joint rotation failed to achieve a significant level but exhibited improvements. This result may be attributable to the small sample; in addition, because the core muscles were healthy, the extent of improvement was limited.

We further investigated the correlation between the maximum achievable angle and the various clinical assessment items, which are presented in Table 4. Among all correlations between the maximum achievable angles and the shoulder-motion angles identified by the CAT, only the maximum angle achieved with shoulder flexion exhibited a significant correlation with shoulder external rotations (ROM); the remaining variables did not exhibit significant correlations. This finding was primarily because when patients engaged in various exercises, they used physical objects, such as resistance bands, towels, and crutches. However, when measuring the ROM, patients did not have any physical objects; thus, the measured shoulder ROM differed, producing no significant correlation. By contrast, only the maximum achievable angle of external rotation (crutch) and the external rotation (muscle strength) exhibited a significant correlation. This result indicated that the increased improvement in external rotation mobility increased the strength of the associated muscles. However, no significant correlations were observed for the other exercises, which was due to the different physical objects used and varying usage habits.

We then investigated the correlations of the number of performed exercises with the various assessment items of the CAT (Table 5). The number of repetitions of shoulder abduction in the scapular plane and shoulder abduction showed significant correlations with the shoulder-motion measurements (i.e., flexion (ROM) and external rotation (ROM)). Thus, we infer that the patient performance in shoulder flexion and external rotation improved by repeating these three exercises.

In summary, four pairs of testing items between CAT and task performance or motor indices were identified to be significantly correlated. This finding implied that the task performance and motor indices were able to detect the therapeutic effects measured by the CATs. Nonetheless, further analysis will be needed to construct more exact relationship between these four pairs of correlated indicators.

Stepwise regression analysis was conducted to investigate the explanatory power of task performance (Tables 6 and 7). The results showed that the total number of movement repetitions of shoulder abduction in the scapular plane effectively predicted the shoulder flexion (ROM). Thus, increasing the number of this motor rehabilitation substantially improved the therapeutic effect of flexion (ROM). In addition, the maximum achievable angle of external rotation (crutch) effectively predicted the external rotation (muscle strength). Thus, increasing the achievable angles during rehabilitation improved the therapeutic effect of external rotation (muscle strength).

Previous studies [13, 14] have integrated VR techniques with motor rehabilitation treatment. These studies used only the CATs to assess the therapeutic effects, neglecting the considerable data collected during the exercises. In this study, we not only used the CATs to assess the therapeutic effects of the exercises but also adopted data derived from the innovative VR rehabilitation system developed in this study to calculate and determine the task performance and motor indices and to evaluate the therapeutic effects. Previously, Pastor et al. [16] based improvements in therapeutic effects on improvements in game performance; however, their assessment results obtained according to the CATs indicated no improvement, impeding the ability of Pastor to assess therapeutic effects. By contrast, the values of task performances and motor indices derived from the GDSR exercise system showed an improvement, demonstrating the consistency with those obtained using the objective CATs. Moreover, by using the correlation analysis, we successfully determined the best task performance items which exhibited a significant correlation with the items of the CATs. Finally, we used stepwise regression analysis and successfully identified the best task performance items which could predict CAT results in order to assess the therapeutic effect.

The study showed that the data collected from the training sessions can be converted into the ingredients related to motor functions and could be used for motor assessment effectively. The clinical evaluation measured by the motor ingredients could be conducted immediately at the end of each training session instead of being performed before and after the training sessions. The motor ingredients examined in this study provided useful and important information for planning better and individualized training programs.

The limitation of our study was the small sample size. A large sample size could reinforce our study results. In addition, the wireless sensors used in our system encountered limitations regarding transmission speed and data retrieval accuracy. Moreover, the effects of size and weight of the wireless sensors on patient joint mobility require clarification.

## 5. Conclusion

In this study, we successfully combined WIMU sensors and interactive VR techniques to develop a novel GDSR exercise system, which could be applied to patients with frozen shoulders as rehabilitation training for shoulder joint mobility and shoulder endurance. We conducted clinical trials on 16 patients and used the motor trajectory of the system to develop the motor indices. By using the motor indices, task performances, and CATs, we statistically confirmed the effectiveness of the GDSR system for frozen shoulder rehabilitation. Moreover, through correlation analysis, we effectively verified the correlation between task performance and CAT items. Finally, we used stepwise regression analysis to identify the best task performance item which could be used to predict the CAT results. This study provided preliminary evidence showing that data collected from WIMU might apply to motor assessment as clinical assessment did. However, further study is needed to figure out how to convert WIMU data to clinical assessment items quantitatively.

## Figures and Tables

**Table 1:** CAT results (*N* = 16).

CATs		Mean	SD	*Z* value	Significance (2-tailed)
CMS	Before	58.4	14.3	3.41	*∗∗*
	After	79.5	9.47		

ROM (degree)					
Flexion	Before	146.5	17.6	3.52	*∗∗*
	After	161.9	13.1		
Abduction	Before	134.5	33.5	3.52	*∗∗*
	After	149.6	26.1		
External rotation	Before	66.4	22.4	3.52	*∗∗*
	After	78.2	16.9		
Internal rotation	Before	49.5	21.3	3.41	*∗∗*
	After	61.1	16.8		

Muscle strength (kg)					
Flexion	Before	16.45	5.47	3.52	*∗∗*
	After	22.3	6.65		
Abduction	Before	14.38	4.52	3.21	*∗∗*
	After	19.23	6.80		
External rotation	Before	14.7	3.39	2.92	*∗∗*
	After	17.6	3.01		
Internal rotation	Before	16.20	7.60	3.35	*∗∗*
	After	21.36	6.87		

Significance level = 0.05.

^*∗∗*^ *P* < 0.01.

**Table 2 tab2:** Task performance results (*N* = 16).

	Exercise		Mean	SD	*Z* value	Significance (2-tailed)
Maximum achievable angles (degree)	Shoulder flexion	1st	136.50	15.59	−3.52	*∗∗*
		8th	160.12	10.65		
	Shoulder abduction in scapular plane	1st	128.93	16.72	−3.52	*∗∗*
		8th	157.00	11.34		
	Shoulder abduction	1st	118.37	30.02	−3.52	*∗∗*
		8th	146.68	26.23		
	Shoulder external rotation	1st	45.56	14.60	−3.41	*∗∗*
		8th	61.93	10.07		
	Shoulder internal rotation (towel)	1st	61.93	19.78	−3.52	*∗∗*
		8th	85.56	15.60		
	Shoulder external rotation (crutch)	1st	49.31	15.87	−3.52	*∗∗*
		8th	67.00	11.15		
	Shoulder external rotation (chair)	1st	86.50	12.82	−3.52	*∗∗*
		8th	110.18	9.07		
	Muscle strength of the abdomen and the front of the thigh	1st	57.06	14.63	−3.30	*∗∗*
		8th	79.06	5.66		
	Muscle strength of the abdomen and the back of the thigh	1st	16.93	5.07	−3.52	*∗∗*
		8th	27.06	5.24		
	Core muscle strengthening	1st	18.68	3.21	−3.47	*∗∗*
		8th	26.62	3.93		

Cumulative number of repetitions of exercises	Shoulder flexion	1st	10.50	2.58	−2.96	*∗∗*
		8th	13.12	1.89		
	Shoulder abduction in scapular plane	1st	10.50	2.58	−2.96	*∗∗*
		8th	13.12	1.89		
	Shoulder abduction	1st	10.50	2.58	−2.97	*∗∗*
		8th	13.06	1.80		
	Shoulder external rotation	1st	9.31	3.35	−2.46	*∗*
		8th	12.06	2.40		
	Shoulder internal rotation (towel)	1st	8.75	3.15	−2.94	*∗∗*
		8th	12.12	1.45		
	Shoulder external rotation (crutch)	1st	9.00	3.30	−2.15	*∗*
		8th	11.18	2.37		
	Shoulder external rotation (chair)	1st	9.00	3.30	−2.00	*∗*
		8th	10.93	2.37		
	Muscle strength of the abdomen and the front of the thigh	1st	8.87	3.13	−2.52	*∗*
		8th	11.06	2.40		
	Muscle strength of the abdomen and the back of the thigh	1st	9.50	2.47	−2.39	*∗*
		8th	11.62	1.66		
	Core muscle strengthening	1st	9.25	3.13	−2.17	*∗*
		8th	11.06	2.40		

Significance level = 0.05.

^*∗*^ *P* < 0.05.

^*∗∗*^ *P* < 0.01.

**Table 3:** Motor indices results (*N* = 16).

	Exercise		Mean	SD	*Z* value	Significance (2-tailed)
Angular velocities (degree/sec)	Shoulder flexion	1st	66.71	35.16	−2.20	*∗*
		8th	81.71	30.57		
	Shoulder abduction in scapular plane	1st	70.76	35.77	−2.10	*∗*
		8th	87.84	38.29		
	Shoulder abduction	1st	48.50	29.59	−2.17	*∗*
		8th	63.07	26.18		
	Shoulder external rotation	1st	62.00	63.77	−2.03	*∗*
		8th	83.57	76.55		
	Shoulder internal rotation (towel)	1st	28.21	14.18	−1.98	*∗*
		8th	42.64	19.27		
	Shoulder external rotation (crutch)	1st	37.37	38.19	−2.38	*∗*
		8th	67.37	44.44		
	Shoulder external rotation (chair)	1st	45.78	26.55	−1.76	0.079
		8th	63.35	23.07		
	Muscle strength of the abdomen and the front of the thigh	1st	62.93	20.76	−2.79	*∗*
		8th	79.86	28.98		
	Muscle strength of the abdomen and the back of the thigh	1st	56.84	17.65	−2.48	*∗*
		8th	88.92	30.26		
	Core muscle strengthening	1st	34.81	29.14	−0.10	0.918
		8th	35.68	26.47		

Number of interruptions as angle increased	Shoulder flexion	1st	1.04	1.54	−1.33	0.182
		8th	0.39	0.55		
	Shoulder abduction in scapular plane	1st	1.44	1.71	−0.08	0.937
		8th	1.17	1.98		
	Shoulder abduction	1st	2.88	3.26	−2.66	*∗∗*
		8th	0.32	0.52		
	Shoulder external rotation	1st	0.31	0.74	0.00	1.000
		8th	0.09	0.22		
	Shoulder internal rotation (towel)	1st	2.35	2.04	−2.69	*∗∗*
		8th	0.48	0.80		
	Shoulder external rotation (crutch)	1st	3.06	8.21	−1.60	0.109
		8th	0.22	0.60		
	Shoulder external rotation (chair)	1st	2.15	2.91	−1.96	*∗*
		8th	0.70	1.14		
	Muscle strength of the abdomen and the front of the thigh	1st	0.28	0.70	−0.42	0.674
		8th	0.29	0.72		
	Muscle strength of the abdomen and the back of the thigh	1st	0.21	0.40	−1.36	0.176
		8th	0.03	0.06		
	Core muscle strengthening	1st	0.68	1.25	−0.53	0.594
		8th	0.80	1.06		

∆F/∆t (kg/sec)	Shoulder flexion	1st	5.57	2.63	−2.98	*∗*
		8th	7.70	2.65		
	Shoulder abduction in scapular plane	1st	6.04	2.82	−2.83	*∗*
		8th	8.29	3.35		
	Shoulder abduction	1st	4.58	2.50	−2.73	*∗*
		8th	6.69	2.46		
	Shoulder external rotation	1st	8.93	9.00	−2.37	*∗*
		8th	15.20	11.95		

Significance level = 0.05.

^*∗*^ *P* < 0.05.

^*∗∗*^ *P* < 0.01.

**Table 4:** Correlations between task performances (angles) and clinical assessments.

CAT	Task performance				
	Shoulder flexion	Shoulder abduction in scapular plane	Shoulder abduction	Shoulder internal rotation (towel)	Shoulder external rotation (crutch)
External rotation (ROM)	0.504 *∗*	0.178 *P* = 0.510	0.102 *P* = 0.707	−0.151 *P* = 0.577	0.465 *P* = 0.070
External rotation (muscle strength)	0.398 *P* = 0.127	0.250 *P* = 0.350	0.366 *P* = 0.163	−0.103 *P* = 0.705	0.497 *∗*

Significance level = 0.05.

^*∗*^ *P* < 0.05 .

Correlation level:

0≤|*r* | < 0.3: low.

0.3 ≤ |*r*| < 0.7: medium.

0.7≤|*r* | < 1: high.

**Table 5:** Correlation between task performances (number of repetitions) and clinical assessments.

CAT	Task performance				
	Shoulder flexion	Shoulder abduction in scapular plane	Shoulder abduction	Shoulder internal rotation (towel)	Shoulder external rotation (crutch)
Flexion (ROM)	0.580 *∗*	0.564 *∗*	0.580 *∗*	0.291 *P* = 0.275	−0.050 *P* = 0.855
External rotation (ROM)	0.514 *∗*	0.514 *∗*	0.524 *∗*	0.233 *P* = 0.385	0.165 *P* = 0.541
Abduction (muscle strength)	−0.158 *P* = 0.560	−0.158 *P* = 0.560	−0.145 *P* = 0.591	−0.507 *∗*	−0.427 *P* = 0.099

Significance level = 0.05.

^*∗*^ *P* < 0.05.

Correlation level:

0≤|*r* | < 0.3: low.

0.3 ≤ |*r*| < 0.7: medium.

0.7≤|*r* | < 1: high.

**Table 6:** Task performance ability to predict flexion (ROM) according to stepwise regression analysis.

Predictor variable	*R* ^2^	Adjusted *R* ^2^	Estimated *β*	*β* coefficient	*t* value	Significance
Shoulder abduction in scapular plane (total number of repetitions)	0.337	0.289	1.559	0.580	2.666	0.018^*∗*^

(a) Predictor variable in the model: shoulder abduction in scapular plane (total number of repetitions).

(b) Dependent variable: flexion (ROM).

^*∗*^ *P* < 0.05.

**Table 7:** Task performance ability to predict external rotation (muscle strength) according to stepwise regression analysis.

Predictor variable	*R* ^2^	Adjusted *R* ^2^	Estimated *β*	*β* coefficient	*t* value	Significance
Shoulder external rotation-crutch (angle)	0.247	0.194	0.131	0.497	2.146	0.050^*∗*^

(a) Predictor variable in the model: (a constant) shoulder external rotation-crutch (angle).

(b) Dependent variable: external rotation (muscle strength).

^*∗*^ *P* < 0.05.
